# Performance of CEUS LI-RADS v2017 major feature combinations: individual patient data meta-analysis

**DOI:** 10.1007/s00261-026-05417-0

**Published:** 2026-02-18

**Authors:** Rawan Awad, Haresh Naringrekar, Robert G. Adamo, Eric Lam, Mostafa Alabousi, Mohammed Kashif Al-Ghita, Stephanie R. Wilson, Jean-Paul Salameh, Mustafa R. Bashir, Andreu F. Costa, Christian B. van der Pol, Eleonora Terzi, Fabio Piscaglia, Bernardo Stefanini, Li-Da Chen, Barbara Meitner-Schellhaas, Deike Strobel, Wei Wang, Xiang Jing, Hyo-Jin Kang, John R. Eisenbrey, Adam Polikoff, Matthew DF McInnes

**Affiliations:** 1 Faculty of Medicine, University of Ottawa, Ottawa, Canada; 2 Department of Radiology, Thomas Jefferson University, Philadelphia, United States; 3 Ottawa Hospital Research Institute, Ottawa Hospital, Ottawa, Canada; 4 William Osler Health System, Brampton, Canada; 5 School of Medicine, Toronto Metropolitan University, Toronto, Canada; 6 Foothills Medical Centre, University of Calgary, Calgary, Canada; 7 Department of Medical Imaging, Ottawa Hospital, Ottawa, Canada; 8 Center for Advanced Magnetic Resonance Development, Duke Medical Center, Durham, United States; 9 Department of Radiology, University of North Carolina at Chapel Hill, Chapel Hill, United States; 10 Department of Diagnostic Radiology, Queen Elizabeth II Health Sciences Centre, Halifax, Canada; 11 Juravinski Hospital and Cancer Centre, McMaster University, Hamilton, Canada; 12 Division of Internal Medicine, Hepatobiliary and Immunoallergic Diseases, IRCSS Azienda Ospedaliero-Universitaria di Bologna, 40138 Bologna, Italy; 13 Department of Medical and Surgical Sciences, University of Bologna, Bologna, Italy; 14 Department of Medical Ultrasonics, Institute of Diagnostic and Interventional Ultrasound, First Affiliated Hospital of Sun Yat-sen University, Guangzhou, China; 15 Department of Internal Medicine, Universitätsklinikum Erlangen, Erlangen, Germany; 16 Department of Ultrasound, Tianjin Institute of Hepatobiliary Disease, Tianjin Third Central Hospital, Tianjin, China; 17 Department of Radiology, Seoul National University Hospital, Seoul, Korea, Republic of

**Keywords:** Contrast enhanced ultrasound (CEUS), LI-RADS, Individual participant data (IPD) meta-analysis, Hepatocellular carcinoma (HCC)

## Abstract

**Background:**

The LI-RADS diagnostic algorithm uses imaging features in contrast-enhanced ultrasound (CEUS) to standardize the diagnosis of hepatocellular carcinoma (HCC) in at-risk patients. However, the diagnostic performance of specific major feature combinations has not been comprehensively evaluated.

**Purpose:**

To evaluate the diagnostic performance of CEUS LI-RADs version 2017 major feature combinations for (HCC) in at-risk individuals across LI-RADS categories 3–5.

**Materials and methods:**

A living systematic review and individual participant data (IPD) meta-analysis was conducted, including studies using CEUS LI-RADS v2016 or v2017 in at-risk patients, identified through database searches updated to February 2024. Eligible observations were categorized per LI-RADS guidelines, and PPVs for HCC were calculated for all major feature combinations in LI-RADS categories 3–5 using a random-effects one-step model. Risk of bias was assessed independently using a customized QUADAS-2 tool.

**Results:**

Thirteen studies were included, comprising 1575 patients (mean age, 62.8 ± 11.2 years; 79.3% male) with 1594 liver observations (median size, 37.1 mm). Pooled PPVs for HCC increased with higher CEUS LI-RADS v2017 categories: LR-3, 40.4% (95% CI: 27.2–55.1); LR-4, 69.7% (95% CI: 49.7–84.3); and LR-5, 95.1% (95% CI: 90.2–97.6). Major feature combinations did not differ from others within the same category. Most studies were at moderate to high risk of bias, primarily due to retrospective design, but sensitivity analysis restricted to low-risk observations yielded similar findings.

**Conclusion:**

CEUS LI-RADS demonstrated progressively higher PPVs from LR-3 to LR-5, supporting its ability to stratify risk across HCC categories. Major feature combinations performed similarly within each category, indicating internal consistency of the system. Although only PPVs were assessed, the results align with trends seen in CT/MRI LI-RADS, with wider confidence intervals in CEUS reflecting smaller sample sizes.

**Supplementary Information:**

The online version contains supplementary material available at 10.1007/s00261-026-05417-0.

## Background

The Liver Imaging Reporting and Data System (LI-RADS) consists of an ultrasound-based screening and surveillance algorithm, diagnostic algorithms using contrast-enhanced ultrasound (CEUS) and CT/MRI, and treatment response algorithms using CT/MRI [1–3]. CEUS examinations are typically applied in problem-solving scenarios to evaluate individual liver observations rather than the whole liver and carries several advantages over CT/MRI. These include a safer contrast agent profile, lack of ionizing radiation, the ability to inject multiple times in the same exam, and higher temporal and spatial resolution with real-time imaging, nearly eliminating the possibility of arterial phase mistiming [3–5]. Liver observations are categorized as LR-1, LR2, LR-3, LR-4 and LR-5, with each LI-RADS category corresponding to a different level of risk for hepatocellular carcinoma (HCC). For CEUS LI-RADS v2017, categorization is determined primarily by three major imaging features: size, presence/absence of non-rim arterial phase hyperenhancement, and type of washout (none, early ≤ 60 s, or late and mild ≥ 60 s) [1].

Prior systematic reviews have focused on the LI-RADS CT/MRI diagnostic algorithm [6–9], with some also including pooled CEUS data [10–12]. Many of these systematic reviews are limited to the study-level, precluding more detailed analyses that would be available at the individual participant or liver observation level. Individual participant data (IPD) meta-analysis, in contrast, involves aggregating more granular data, enabling higher-level analyses by considering the features of each liver observation [13–15]. Prior IPD meta-analyses have explored the probability of HCC for each CEUS LI-RADS major feature and the impact of reference standard on LI-RADS category performance, however, determining the likelihood of HCC using each combination of CEUS LI-RADS major features has not been assessed [16, 17]. Although LI-RADS categories are defined by major features, observations within the same category may reach that designation through different combinations of major features, lesion sizes, and enhancement behaviour. These distinct feature pathways may not carry equivalent malignancy risk, but this potential heterogeneity is obscured when all observations within a category are pooled. Prior work by Adamo et al. identified several outlier observations within LR-3, LR-4, and LR-5 categories for CT/MRI, highlighting that certain feature combinations may deviate from the expected risk profiles [18]. Evaluating major feature combinations allows for assessment of potential within-category variability that is not captured through category-level analysis alone. Such an analysis could validate the CEUS Diagnostic Table and potentially identify areas for improvement [3].

The purpose of this study was to establish the positive predictive value (PPV), of HCC using the various combinations of CEUS LI-RADS v2017 major features in individuals at risk for HCC, for LI-RADS categories 3–5.

## Methods

An ongoing ‘living’ systematic review and individual participant data (IPD) meta-analysis project is underway to evaluate LI-RADS (https://osf.io/tdv7j). This research program continuously reviews the literature for published studies and incorporates new data into the IPD database. Previous studies utilizing the LI-RADS IPD database have been published [6, 16–26]. The current study explores a research question distinct from prior studies, incorporates data from an updated literature search, and employs unique inclusion criteria, analytical approaches, and outcome measures.

The study protocol was reviewed and approved by the institutional review board (IRB) at (Anonymized Hospital Name). No personal health information was shared with the project team. Methodologic guidance was per best practices in diagnostic test accuracy systematic reviews [27, 28]. Reporting was conducted as per the Preferred Reporting Items for a Systematic Review and Meta-analysis of Individual Participant Data (PRISMA-IPD) and PRISMA Diagnostic Test Accuracy (PRISMA-DTA) statements [29–31]. Data was collected and analyzed on a per-observation basis.

### Database search and study selection

With the assistance of an experienced hospital librarian, a search of MEDLINE, Embase, Cochrane Central Register of Controlled Trials (CENTRAL), and Scopus databases was performed for studies evaluating the diagnostic accuracy CEUS for HCC using LI-RADS (v2016 or v2017), updated to February 2024. No language or publication type restrictions were applied. The corresponding authors of each study identified for inclusion were contacted.

### Eligibility criteria

All CEUS studies reporting the percentage of HCC for CEUS LI-RADS categories 1 to 5 in patients at high risk of HCC and eligible for application of LI-RADS (cirrhosis not due to a vascular disorder such as Budd-Chiari syndrome or treatment for hypoplastic left heart syndrome; chronic hepatitis B viral infection; current or prior history of HCC) were eligible for inclusion. Data from studies that did not adhere to LI-RADS CEUS technical guidelines were excluded, for example studies using Kupffer-cell agents (Sonazoid) or a mechanical index ≥ 0.3. All liver observations were required to have been categorized using CEUS LI-RADS v2016/v2017. A preferred composite reference standard was established to assess bias risk [16].

### Data collection process and definitions for data extraction

Authors who did not respond to the invitation to collaborate were sent follow-up emails to maximize sample size. All authors agreeing to participate were sent a formal confidentiality agreement explaining that data will be stored securely and only accessed by authorized co-investigators, as well as a copy of the data contribution form, data extraction sheet, data dictionary, and a list of frequently asked questions. The request for de-identified data included instructions to transfer the information to an encrypted directory. When necessary, data sharing agreements were obtained based on institutional policies. Efforts were made to keep all collaborators involved and informed of progress. Individual participant data were not distributed elsewhere.

### Data validation

IPD were entered and consolidated into a master dataset using an encrypted Research Electronic Data Capture (REDCap) database, with each liver observation assigned a unique identifier [32, 33]. Individual participant data (IPD) provided by the primary study investigators was cross-checked against published reports for validation and positivity rates by a research Methodologist (Anonymized Author), a second-year medical student (Anonymized Author), and a board-certified abdominal radiologist with 8 years of experience using CEUS LI-RADS (Anonymized Author). In cases of unclear or inconsistent data, the primary study investigators were contacted to resolve discrepancies. If multiple readers assessed the same observation and no consensus interpretation was available, data from one reader was randomly selected. LI-RADS major features were classified as either present or absent. Observations lacking information on the presence or absence of any major feature were excluded from analysis.

### Risk of bias and applicability

A previously customized QUADAS-2 tool for application to LI-RADS was used, Appendix S1 [16]. QUADAS-2 divides sources of bias into four categories, namely patient selection, index test, reference standard and flow and timing. Incomplete reporting of major features was flagged using QUADAS-2 under the flow and timing domain. Risk of bias (RoB) and applicability assessment was performed in duplicate, independently, by (Anonymized Author) and one of three other authors (Anonymized Authors), based primarily on the study publication. Differences were resolved by discussion with a third author (Anonymized Author). All RoB assessors conducted a pilot of one study with subsequent discussion to improve inter-observer agreement. While sources of bias for the patient selection domain are generally applicable at the study-level, sources of bias at the index test, reference standard and flow and timing domain could be specific to individual participants and liver observations within the same study. Liver observations or patients within the same study were differentiated by RoB and the relevant domain was flagged on the QUADAS-2 datasheet with explanatory notes to inform administrators of the IPD database. This was performed by following the LI-RADS IPD Group protocol on RoB assessment of individual observations and patients (available at Anonymized Link).

### Outcome measures

For each CEUS LI-RADS v2017 major feature combination, the primary outcome measure was the PPV of HCC observations with 95% CI. PPV represents the likelihood that an observation is an HCC.

### Statistical analysis

To compute the PPV of HCC for each CEUS LI-RADS v2017 major feature combination, the IPD was combined across studies and modelled simultaneously for each major feature combination in LI-RADS categories LR-3, LR-4, and LR-5 using a random-effects model and a one-step approach. Data was assessed on a per-observation basis. PPV for HCC for all major feature combinations was calculated with 95% CIs and depicted visually using forest plots. When model convergence was obtained, clustering was considered at both the patient and study levels. Variability within each cell of the LI-RADS diagnostic table was determined using I^2^ to evaluate between study variance [27]. A chi-square test for the given proportions of HCC was used to test whether there are differences between major feature combinations within each LI-RADS category. For any significant differences in PPV found within a certain LI-RADS category, a Wald test was performed comparing each major feature combination’s random effects model to the random effects model of all other observations in the same LI-RADS category. All analyses were performed using the *glmer*, *metaprop*, and *rma* functions in the *lme4*, *meta*, and *metafor* packages in R [34]. Statistical significance was defined as two-tailed *p* < 0.05.

### Publication bias

Publication bias was not assessed as per contemporary guidance for DTA systematic reviews.

## Results

### Study selection and characteristics

**Fig. 1:**
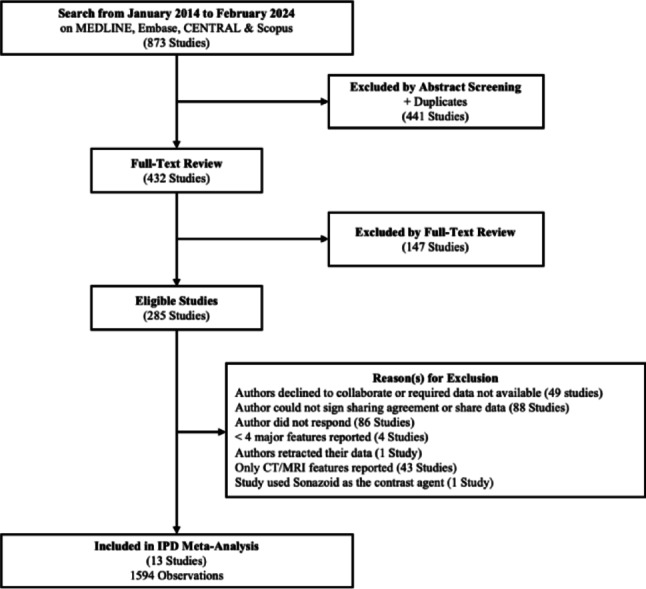
The flow diagram illustrates the Preferred Reporting Items for a Systematic Review and Meta-Analysis (PRISMA), summarizing the search results, study review, and exclusion criteria for all studies. Of the 873 studies identified, 13 met the inclusion criteria and were included in the individual participant data (IPD) meta-analysis, encompassing 1594 liver observations

Study screening, inclusion, and exclusion criteria are depicted using a flow diagram, presented in Fig. 1. Thirteen studies were included consisting of 1575 patients (mean age, 62.8 years ±11.2 [SD]; 79.3% male patients [1249 of 1575]; age range, 7–88 years; IQR, 56–70 years) with 1594 liver observations (median size, 37.1 mm). Study characteristics are presented in Tables 1 and 2. The prevalence of overall HCC, malignancy, and benignity of liver observations for the study population are presented in Table 3.

**Table 1:** Study details of included studies

Study	Country	Study design	Prevailing risk factor	Modality	Role of CEUS in diagnostic pathway	Contrast agent	LI-RADS version	No. of readers
Chen [39]	China	RCCon	HBV with cirrhosis	CEUS	Primary CEUS surveillance	Blood pool	2017	2
Chen [40]	China	RC	HBV > Cirrhosis	CEUS	Primary CEUS surveillance	Blood pool	2017	2
Ding [41]	China	RC	Cirrhosis > HBV	CT, MRI, CEUS	Primary CEUS surveillance	ECA, HPB, Blood pool	2017	2
Ding [42]	China	RC	Cirrhosis > HBV	CEUS	Primary CEUS surveillance	Blood pool	2017	2
Hu [43]	Canada	PC	Cirrhosis > > HBV	CEUS, MRI	MRI indeterminate, then CEUS	Blood pool, ECA & HPB	2017, 2018	2
Kang [44]	South Korea	PC	HBV > Cirrhosis	CT, MRI, CEUS	CT/MRI indeterminate, then CEUS	ECA, HPB, Blood pool	2017, 2018	2
Makoyeva [45]	Canada	RC	Cirrhosis > > HBV	CEUS	MRI indeterminate, then CEUS	Blood pool	2016	3
Mulazzani [46]	Italy	PC	Cirrhosis > > HBV	CEUS	Primary CEUS surveillance	Blood pool	2017	NR
Pan [47]	China	RC	HBV > Cirrhosis	CEUS	CEUS surveillance only	Blood pool	2017	2
Polikoff [48]	USA	RC	Cirrhosis ~ HBV	CT, MRI, CEUS	Primary CEUS surveillance	ECA, HPB, Blood pool	2017, 2018	2
Strobel [36]	Germany	PC	Cirrhosis > > HBV	CEUS	Primary CEUS surveillance	Blood pool	2013	50
Terzi [49]	Italy	RC	Cirrhosis > > HBV	CEUS	Primary CEUS surveillance	Blood pool	2017	NR
Zhou [50]	China	RC	HBV > Cirrhosis	CT, MRI, CEUS	Primary CEUS surveillance	ECA, HPB, Blood pool	2017, 2018	2

Most studies were retrospective cohorts from Asia, primarily in patients with cirrhosis. The most used modality was contrast-enhanced ultrasound (CEUS) with a blood pool contrast agent. In most studies, CEUS served as the primary surveillance modality rather than a secondary examination after inconclusive CT/MRI. LI-RADS version 2017 was the most frequently used classification system. Symbols are defined as follows: ~ indicates that both risk factors were represented approximately equally; > indicates that one risk factor is larger than the second risk factor; and > > indicates that the first risk factor was represented substantially more in the cohort than the second risk factor, defined as at least twice as common (i.e. a 2:1 ratio). *CEUS* contrast-enhances US, *ECA* extracellular contrast agent, *HBV* hepatitis B virus, *HPB* hepatobiliary contrast agent, *NR* not recorded, *PC* prospective cohort, *RC* retrospective cohort, *RCCon* retrospective case control, *USA* United States of America

**Table 2:** Observation data of included studies

Study	No. of liver observations/patients	No. of HCC/ observations (%)	No. Malignancy	No. Benign	No. LR-1	No. LR-2	No. LR-3	No. LR-4	No. LR-5	No. LR-TIV/5	No. LR-M	Reference standard
Chen [39]	176/176	88 (50)	176	0	NR	NR	1	6	49	0	120	P
Chen [40]	210/105	105 (50)	210	0	NR	NR	1	7	64	0	138	P
Ding [41]	273/239	225 (82)	22	26	NR	NR	16	25	173	0	59	P
Ding [42]	264/264	223 (84)	23	18	NR	NR	6	18	145	0	95	P
Hu [43]	39/35 38/34	11 (28) 11 (29)	12 12	27 26	22 NR	1 NR	4 NR	1 NR	10 NR	0 NR	1 NR	P and CCRS
Kang [44]	CEUS 43 CT 35 MRI 8	CEUS 20/43 (47) CT 16/35 (46) MRI 4/8 (50)	CEUS 21 CT 17 MRI 4	CEUS 0 CT 18 MRI 4	CEUS 16 CT NR MRI NR	CEUS 23 CT NR MRI NR	CEUS 21 CT NR MRI NR	CEUS 22 CT NR MRI NR	CEUS 31 CT NR MRI NR	CT NR MRI NR CEUS0	CT NR MRI NR CEUS 2	P and CCRS
Makoyeva [45]	196/184	139 (71)	157	39	10	1	24	8	116	8	29	P and CCRS
Mulazzani [46]	54/34	33 (61)	34	20	6	3	4	7	25	3	1	P and CCRS
Pan [47]	545//534	503 (92)	29	13	0	0	11	22	294	0	218	P
Polikoff A 2022 [48]	50/29	50/50 (100)	0	0	CT 0 MRI 0 CEUS 0	CT 0 MRI 0 CEUS 0	CT 0 MRI 0 CEUS 0	CT 1 MRI 18 CEUS 7	CT 0 MRI 20 CEUS 17	CT 0 MRI 0 CEUS 0	CT 0 MRI 0 CEUS 0	P
Strobel [36]	470/470	378 (80)	43	49	NR	NR	NR	77	197	NR	40	P and CCRS
Terzi [49]	333/NR	278 (83)	289	44	0	0	74	97	144	0	18	P and CCRS
Zhou [50]	213/213	180 (85)	8	25	0	0	16	29	135	0	33	P

Most studies reported a high proportion of hepatocellular carcinoma (HCC) observations, with LI-RADS 5 being the most frequently assigned category. Contrast-enhanced ultrasound (CEUS) was the primary imaging modality, and pathology (P) was the most used reference standard. *CCRS* composite reference standard, *NR* not recorded

**Table 3:** Patient characteristics for CEUS liver imaging and data system categories

Parameter	Total
No. of patients	1575
No. of observations	1594
Mean age (years)	62.8 ± 11.2
Female patients (%)*	316 (20.1)
Male patients (%)*	1249 (79.3)
Prevalence of HCC (%)	1349 (84.6)
Prevalence of overall malignancy (%)	1385 (86.9)
Prevalence of benign lesions (%)	152 (9.5)
Prevalence of lesions defined as “no HCC” or “other” (%)^†^	57 (3.6)

Data in parentheses are percentages. Mean data are ± SDs. Most patients included in this study were male with a mean age of 62.8 years. Hepatocellular carcinoma (HCC) was the most common diagnosis overall (84.6%). Lesions defined as “no HCC” or “other” accounted for 3.6% of observations

*Patient sex was not available for 10 liver observations (6 patients)

^†^Lesions that were reported as “None” (HCC) or “Other (Specify)” in the IPD

### Risk of bias and applicability

**Fig. 2:**
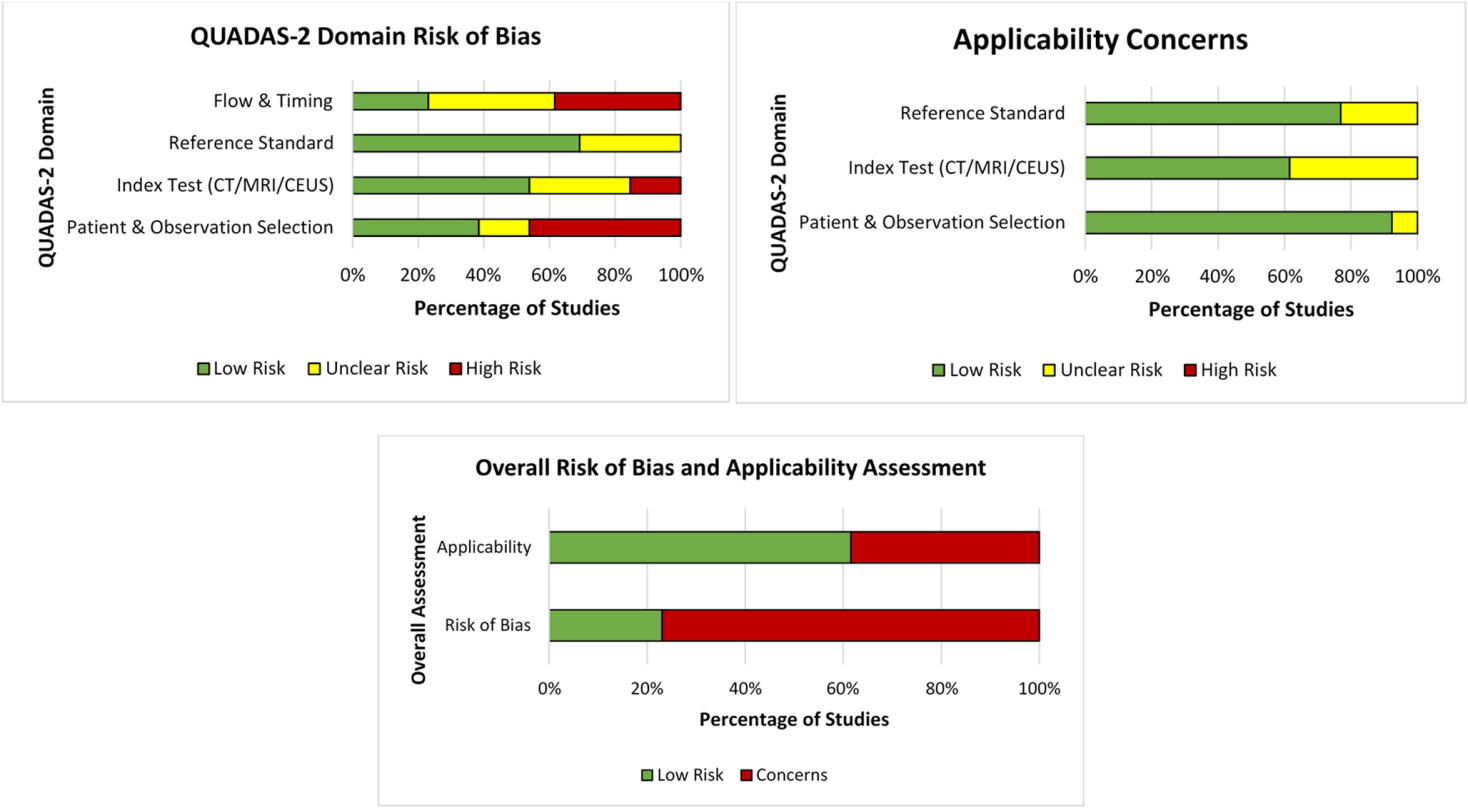
Bar plots show the results of the Quality Assessment of Diagnostic Accuracy Studies 2 (QUADAS-2) risk of bias (RoB) assessment. Thirteen studies were included in the assessment. Most studies showed low RoB at the reference standard and index test domains, however, flow and timing and patient and observation selection domains demonstrated higher RoB (top left panel). Most studies demonstrated low applicability concerns at the reference standard, index test, and patient and observation selection applicability concerns (top right panel). Overall, RoB was high in most studies, while applicability concerns were generally low across studies (bottom panel)

Of the 13 studies included, 23% (3 of 13) were at low RoB and 62% (8 of 13) had low concerns about applicability (Fig. 2). The QUADAS-2 ratings per study are presented in Table S1. Patient and observation selection were the most common domains at high RoB (46% of studies; 6 of 13), due to retrospective designs, lack of consecutive or random sampling, or exclusion of non-HCC lesions. The second most common domain at high RoB was flow and timing (38% of studies; 5 of 13), often due to incomplete inclusion of eligible patients or insufficient reporting on the interval between CEUS and the reference standard. The index tests domains were at high RoB in 15% of studies (2 of 13), due to either the CEUS readers not being blinded to clinical/outcome information or not being clearly reported. The reference standard domains were at low RoB for most of the included studies (69%; 9 of 13).

### PPV of CEUS major feature combination

**Fig. 3:**
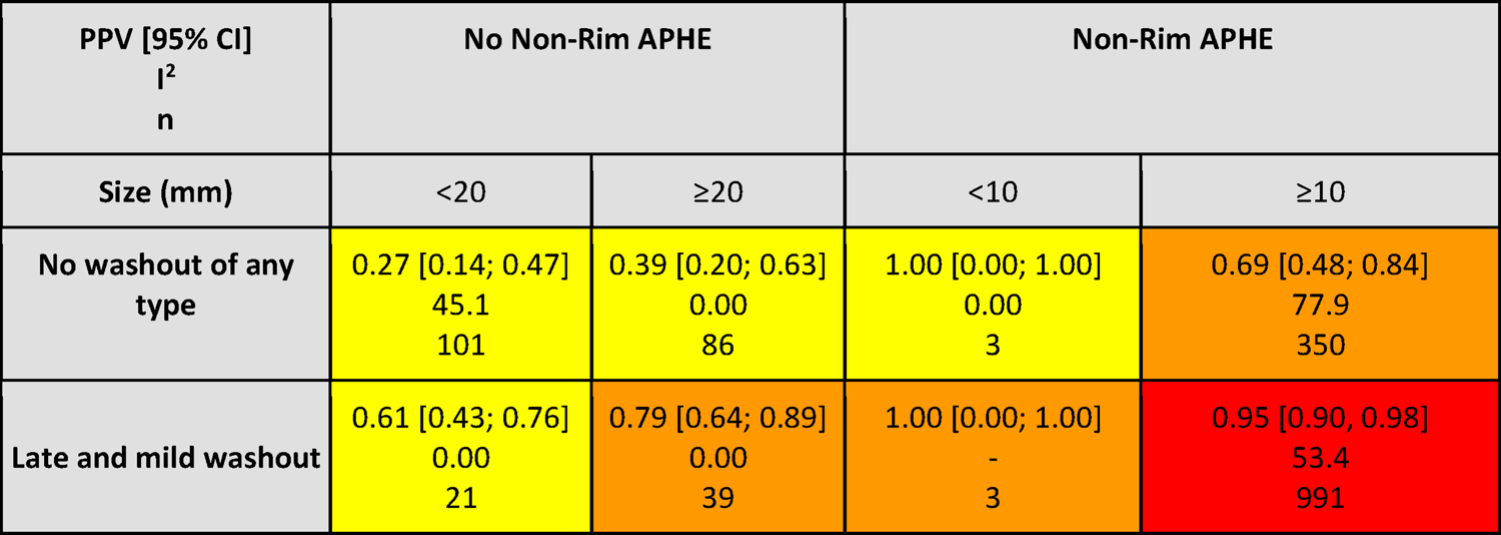
Diagnostic table shows the random-effects model of positive predictive values (PPVs) for diagnosis of hepatocellular carcinoma (HCC) for contrast-enhanced ultrasound (CEUS) Liver Imaging Reporting and Data Systems (LI-RADS) major feature combinations. The pooled PPV estimates and 95% CIs for each LI-RADS category were as follows: LR-3, 40.4% (95% CI: 27.2–55.1); LR-4, 69.7% (95% CI: 49.7–84.3); LR-5, 95.1% (95% CI: 90.2–97.6). Each cell in the diagnostic table shows PPVs at the top and I^2^ values in the middle, which indicate diagnostic performance and between-study heterogeneity, respectively. Each cell also shows the sample size (n) at the bottom. Data in parentheses are 95% CIs. PPVs increased with observation size (< 10 mm to ≥ 10 mm), presence of arterial phase hyperenhancement (APHE), and the presence of late and mild washout. LR-3 combinations (yellow) showed the lowest PPVs and often low heterogeneity (I^2^), LR-4 combinations (orange) showed intermediate PPVs with variable heterogeneity, whereas LR-5 combinations (red) demonstrated consistently high PPVs with generally high I^2^ values

**Fig. 4:**
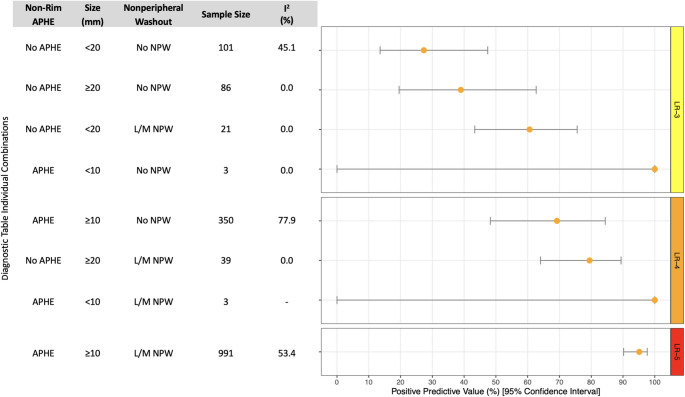
Random-effects model shows positive predictive values (PPVs) for diagnosis of hepatocellular carcinoma (HCC) for CEUS Liver Imaging Reporting and Data Systems (LI-RADS) major feature combinations compared with the LI-RADS grade overall PPV without the combination of interest. The individual feature combinations for the LI-RADS diagnostic table (left side) shows observation size (< 10 mm, ≥ 10 mm, < 20 mm, and ≥ 20 mm), arterial phase hyperenhancement (APHE), and non-peripheral washout (NPW; no NPW or late/mild (L/M) NPW). Between-study heterogeneity is quantified using I^2^, which reflects the proportion of variability due to heterogeneity rather than chance. The forest plot (right side) shows the PPVs (orange dots) with 95% CIs (gray lines). Combinations are grouped by LI-RADS category: LR-3 (yellow), LR-4 (orange), LR-5 (red). A total of 1594 observations were included

The CEUS diagnostic table showing the PPV for HCC for major feature combinations grouped according to LI-RADS v2017 is presented in Fig. 3 and Table S2. The pooled PPV for LR-3 was 40.4% (95% CI: 27.2–55.1), for LR-4 was 69.7% (95% CI: 49.7–84.3), and for LR-5 was 95.1% (95% CI: 90.2–97.6). I² values were generally low across analyses, with only two combinations showing substantial heterogeneity: the LR-4 combination of APHE ≥ 10 mm with no washout (I^2^ = 77.9%), and the LR-5 combination of APHE ≥ 10 mm with late/mild washout (I^2^ = 53.4%) (Figs. 3 and 4). All other combinations demonstrated I^2^ = 0%, except for one LR-3 combination (no APHE, < 20 mm with no washout) which showed I^2^ = 45.1%, and one LR-4 combination (APHE < 10 mm with late/mild washout) which had insufficient data to estimate I^2^ (*n* = 3).

Within-category analyses showed significant variation in PPVs among major feature combinations in LR-3 (χ² *p* = 0.021) and LR-4 (χ² *p* = 0.022) (Table S4). There were no comparisons performed within LR-5 for CEUS as only observations ≥ 10 mm with APHE and late mild washout fall into this category.

In the LR-3 and LR-4 categories for CEUS LI-RADS, none of the individual major feature combinations differed significantly from the category’s overall pooled PPV (Figs. 3 and 4; Table S2).

### Sensitivity analysis

A sensitivity analysis restricted to observations with low RoB found the population to have wider 95% CIs due to reduced sample size, limiting comparability between categories. All feature combination PPVs had 95% CIs overlapping with other feature combinations in the same category. There was one notable finding within LR-3: the combination defined by no APHE, lesion size < 20 mm, and no washout had a PPV of 7.9% (95% CI: 0.2–83.0). Although this overlapped with the overall LR-3 PPV estimate (40.4%; 95% CI: 27.2–55.1), it was markedly less precise than in the primary analysis (27.4%; 95% CI: 13.6–47.4) (Figs. 3 and 4; Table S2; Figure S2). However, Wald tests performed in the sensitivity analysis showed that the individual combinations for PPVs for LR-3 were not significantly different from the overall LR-3 PPV, consistent with the main results where sample size permitted testing (Table S3). Statistical analysis within several subgroups could not be performed due to the small sample size.

## Discussion

This individual participant data (IPD) meta-analysis of 13 studies comprising 1594 liver observations aimed to evaluate the performance of CEUS Liver Imaging Reporting and Data (LI-RADS) v2017 by assessing the positive predictive values (PPVs) of major feature combinations in patients at high risk for hepatocellular carcinoma (HCC). We found that positive predictive values (PPVs) increased progressively across LI-RADS categories, from 40.4% (95% CI: 27.2–55.1) for LR-3, to 69.7% (95% CI: 49.7–84.3) for LR-4, and 95.1% (95% CI: 90.2–97.6) for LR-5. Furthermore, all combinations within each LR-3, LR-4, and LR-5 category had overlapping 95% CIs, supporting the reliability of CEUS LI-RADS for risk stratification and clinical decision making in at-risk populations. However, two combinations, LR-3 (APHE, < 10 mm without washout) and LR-4 (APHE, < 10 mm with late/mild washout), had very small sample sizes (both *n* = 3), limiting the precision of PPV estimates and hindering reliable assessment of those specific cells.

Our results are consistent with previous systematic reviews and meta-analyses of CEUS LI-RADS, which also reported increasing probabilities of HCC from LR-3 to LR-5 [10–12]. While this overall pattern is expected given the design of the LI-RADS system, the primary purpose of the present analysis was not to confirm increasing malignancy risk from LR-3 to LR-5, but rather to determine whether category-level outliers exist within CEUS LI-RADS similar to those previously identified for CT/MRI LI-RADS [18]. By evaluating diagnostic performance at the level of CEUS major-feature combinations, this study demonstrates an absence of such outliers. These findings suggest strong internal consistency within CEUS LI-RADS categories, indicating that no revisions to the current CEUS framework are necessary based on the available evidence.

**Fig. 5:**
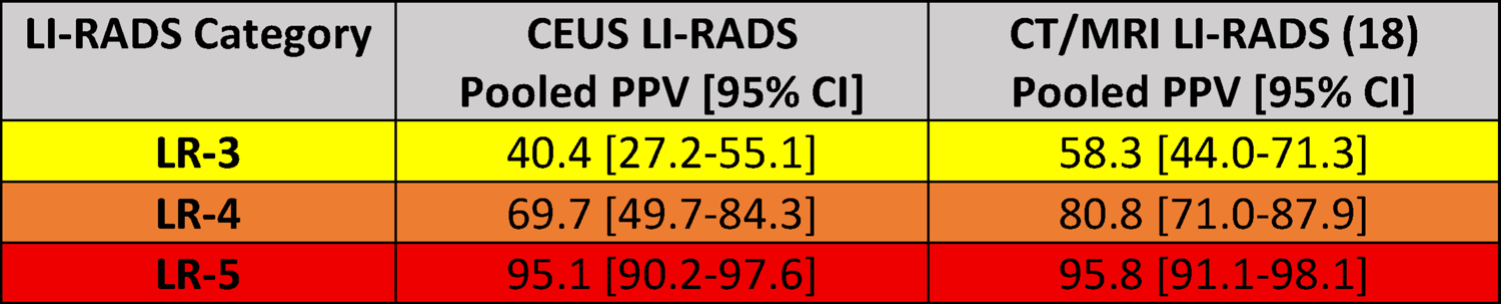
Comparison of pooled positive predictive value (PPV) estimates for LI-RADS categories LR-3, LR-4, and LR-5 between CEUS LI-RADS (current study) and CT/MRI LI-RADS as reported by Adamo et al. [18]. Each cell presents the pooled PPV estimates with the corresponding 95% confidence intervals (CIs) in parentheses

When compared with the recently published CT/MRI IPD meta-analysis of LI-RADS major feature combinations [18], our findings demonstrate similar diagnostic stratification across categories. CEUS and CT/MRI yielded relatively consistent PPVs for LR-3 (40.4% vs. 58.3%) and LR-4 (69.7% vs. 80.8%), and both showed excellent performance for LR-5 (95.1% vs. 95.8%), as shown in Fig. 5. These similarities suggest that LI-RADS major feature definitions stratify HCC risk consistently across modalities, underscoring the strength of the framework. Small differences observed between CEUS and CT/MRI may reflect technical factors such as temporal resolution or vascular characterization, as well as population differences between included studies [6–10, 16, 18]. An important difference between the CEUS and CT/MRI results is in the precision of PPVs because of lower sample sizes of the CEUS IPD.

In examining the design characteristics of the included studies, most used CEUS as the initial surveillance modality rather than as a secondary problem-solving test following indeterminate CT or MRI findings (Table 1). Only three studies evaluated CEUS after indeterminate CT/MRI, while the remainder applied CEUS as the primary imaging test. This distinction is relevant to interpreting diagnostic performance, as studies using CEUS in a troubleshooting context may include a higher proportion of atypical or indeterminate lesions, raising the pre-test probability of malignancy and potentially inflating PPVs relative to studies in which CEUS was performed as first-line surveillance. In contrast, studies where CEUS was performed as the initial test may reflect broader real-world surveillance populations. This variation in imaging pathways highlights the importance of considering differences in clinical context and referral populations when comparing CEUS and CT/MRI performance.

Between-study heterogeneity was concentrated in a few combinations, particularly within LR-4 and LR-5. I^2^ represents the proportion of total variation in effect estimates that is due to true differences between studies rather than chance, with higher values indicating greater inconsistency across study results, potentially due to variability in study populations, methods, or imaging protocols. Substantial heterogeneity (I^2^ ≥ 50%) was observed for the LR-4 combination of APHE ≥ 10 mm with no washout (I^2^ = 77.9%), and the LR-5 combination of APHE ≥ 10 mm with late/mild washout (I^2^ = 53.4%). These combinations represented the largest subgroups, with 350 and 991 observations, respectively. The remainder of the combinations demonstrated I^2^ = 0%, excluding one LR-3 combination (no APHE, < 20 mm with no washout) which showed I^2^ = 45.1%, and one LR-4 combination (APHE, < 10 mm with late/mild washout) which had insufficient data to estimate I^2^. Comparable findings were reported in Adamo et al.’s CT/MRI IPD meta-analysis (2025), where analogous LR-4 and LR-5 combinations also demonstrated substantial heterogeneity of 81.8% and 54.9% respectively [18], suggesting that these patterns may reflect a broader reproducibility challenge across modalities. Importantly, restricting analyses to studies at low RoB virtually eliminated heterogeneity, indicating that methodological quality may be a major contributor. In addition to these design factors, several CEUS-specific technical and interpretive elements may also contribute to the higher heterogeneity seen in the largest LR-4 and LR-5 subgroups. Several technical parameters are recommended to preserve microbubbles, including using a low mechanical index (< 0.3), having an adequate IV line for injection (18–22 gauge) using a 3-way stop-cock, hand injection followed by saline flush, and intermittent imaging after peak APHE. Differences in microbubble injection and handling can therefore affect bubble stability, arterial enhancement appearance, and the duration of visible contrast, which may in turn influence the assessment of APHE and late/mild washout [4, 5, 35, 36]. Interpretative variation may also arise from reader experience with CEUS LI-RADS, particularly in borderline cases where the threshold for calling APHE or late/mild washout differ across centers [35, 36]. In contrast to the more standardized acquisition environment of CT/MRI, the dynamic real-time nature of CEUS and its greater dependence on operator technique may amplify between-study variability. These findings suggest that heterogeneity in CEUS may not be a global-category-level effect but arises from the combination of study quality, case mix, and modality-specific technical factors. Future studies may benefit from emphasizing standardized CEUS acquisition, rigorous reader training, and multicenter prospective designs with adequate per-cell sample sizes to improve precision and reduce variability.

Risk of bias (RoB) assessments indicated that only 23% of the included studies were at low RoB, with patient observation and selection and flow and timing being the most frequent high RoB domains. These domain-level issues align with known challenges in imaging accuracy research, including retrospective study designs, non-consecutive or convenience sampling, incomplete inclusion of eligible observations, and variable or insufficiently reported intervals between CEUS and the reference standard. These factors may limit the generalizability to study populations and could influence the observed PPVs within individual LI-RADS categories. This pattern of high-risk domains is consistent with prior QUADAS-2 evaluations of RoB and applicability across LI-RADS-related publications and highlights the value of prospective designs with consecutive enrollment and clearly reported intervals between CEUS and the reference standard [24]. In the low RoB sensitivity analysis, the PPV values were similar to the main analyses, but less precise due to smaller sample sizes. For example, in the LR-3 combination of no APHE, < 20 mm, and no washout, the PPV decreased from 27.4% (95% CI: 13.6–47.4) in the primary analysis to 7.9% (95% CI: 0.2–83.0) in the low RoB subset, illustrating the wide uncertainty from sparse data. For the single LR-5 combination (APHE, ≥ 10 mm, late/mild washout), the low-RoB PPV remained high at 97.1% (95% CI: 93.6–98.7). Several subgroup comparisons could not be performed, and we did not detect additional LR-3 outliers on Wald testing, which is consistent with low power given the broad confidence intervals. Although the sensitivity analysis supports the main findings, it contributes limited confirmatory weight due to the small sample size and should be interpreted with appropriate caution given the underlying study quality and limited confirmatory weight of the low-RoB subset.

A significant limitation of this study is the small sample sizes for several feature combinations, especially in the LR-3 and LR-4 categories (non-rim APHE < 10 mm), yielding wide CIs and limiting power for within-category comparisons. CEUS LI-RADS 2017 currently requires liver lesions to be visible on pre-contrast gray scale imaging, which may be challenging to identify for these combinations due to the small observation size. CEUS microbubble specific factors may also contribute to the small sample size for these feature combinations as CEUS contrast is purely intravascular with high sensitivity on ultrasound allowing for rapid visualization of hepatic arteries and parenchymal enhancement. This may contribute to reduced identification of vascular shunts that are often categorized as LR-3 on CT/MRI [37]. Finally, the relatively small number of observations in these LR-3 and LR-4 combinations with lower lesion size cutoffs (< 10 mm) compared with larger sample sizes observed in LR-4 and LR-5 combinations with larger size cutoffs (≥ 10 mm) likely contributed to a larger median lesion size of 37 mm across included observations. This may partly contribute to higher pooled PPV estimates, particularly in the LR-4 category, relative to clinical settings in which smaller nodules may be more commonly encountered.

The relative scarcity of CEUS studies compared with CT/MRI likely reflects practical barriers such as the need for specialized ultrasound equipment, lack of operator training, and variable familiarity with CEUS, which may also contribute to center-level variability. Furthermore, CEUS may yield fewer observations characterized by APHE-only or washout-only features compared to CT/MRI. On CT/MRI, washout may reflect relative parenchymal enhancement on delayed phases rather than true contrast decline, and APHE can include perfusional shunts or pseudo-lesions that are not true lesions. By contrast, CEUS requires a visible observation on pre-contrast grayscale imaging, and both APHE and washout must represent true enhancement dynamics, making pseudo-lesions rare [35]. These modality-specific factors may contribute to the lower number of observations in certain CEUS algorithm feature combinations relative to CT/MRI. As such, these considerations should inform study design and training priorities for future CEUS studies.

Another limitation of this study is the geographic and etiologic composition of the included patients. Most studies were conducted in East Asia, particularly in China, and most study populations had HBV-related chronic liver disease or cirrhosis (Table 1). Although current evidence does not strongly support meaningful differences in LI-RADS diagnostic performance across geographic regions or underlying etiologies of chronic liver disease, the predominance of HBV-related cohorts may limit generalizability to regions where MAFLD/MASH or alcohol-related liver diseases are more common. Prior work suggests that LI-RADS maintains comparable performance across etiologic contexts, supporting its broad applicability [38]. However, future studies with greater representation of MAFLD/MASH and other non-HBV predominant populations may be valuable to confirm whether the observed PPV trends for CEUS LI-RADS major feature combinations remain consistent across diverse clinical settings.

In conclusion, CEUS LI-RADS major feature combinations show within-category consistency, with successively increasing PPVs from LR-3 to LR-5, and PPVs which are comparable to CT/MRI. Heterogeneity was concentrated in specific combinations and was reduced in analyses restricted to low RoB studies, suggesting that methodological quality and technical factors may play a larger role than global category-level effects. Given the limited precision of the low RoB subset, sensitivity analysis findings are supportive but not definitive. However, our results reinforce the utility of CEUS LI-RADS for clinical risk stratification of HCC.

## Supplementary Information

Below is the link to the electronic supplementary material.

Supplementary Material 1

## Data Availability

The individual participant data (IPD) used in this study were obtained directly from primary study investigators as part of the ongoing LI-RADS Individual Participant Data (IPD) Meta-Analysis living systematic review project, registered at https://osf.io/tdv7j. Data were shared under institutional data-sharing and confidentiality agreements, in accordance with local ethics and privacy regulations, and stored securely within an encrypted Research Electronic Data Capture (REDCap) database. Because these datasets contain potentially identifying information from multiple institutions, they are not publicly available. De-identified, aggregated data supporting the results of this study are available from the corresponding author upon reasonable request and with permission from the contributing institutions.
