# Supplementary material for: Performance of CEUS LI-RADS v2017 major feature combinations: individual patient data meta-analysis
Source: Abdom Radiol (NY). 2026 Feb 18;51(8):3957–70. doi: 10.1007/s00261-026-05417-0 (PMC13388517; doi:10.1007/s00261-026-05417-0)
Supplement: Supplementary file 1 — Supplementary Material 1 [file 261_2026_5417_MOESM1_ESM.docx]

**Appendix S1.** Reference Standard

A preferred reference standard was established to assess the RoB for each liver observation as used in prior works (16). For HCC, histopathology from core needle biopsy, hepatectomy or explanation was favored. Otherwise, a composite clinical reference standard was used. An observation was considered benign if it was stable on imaging for at least 12 months, or if it demonstrated either an unequivocal spontaneous size reduction of at least 30% diameter or disappearance in absence of treatment, not attributable to resorption of tumoral blood products. An observation was considered positive for HCC if it fulfilled LR-5 criteria on another contrast enhanced imaging modality and showed threshold growth (≥50% size increase in less than 6 months); or was categorized LR-5 then underwent locoregional treatment and recurred on CT or MRI based on the LI-RADS treatment response criteria. All other malignancies required histopathology for confirmation. LR-3, LR-4 and LR-M observations with recurrence on CT or MRI after local treatment were considered malignant but not specifically HCC.

**Table S1.** QUADAS-2 ratings for each primary study included in the present study.

| **Study** | **Domain 1: Patient and Observation Selection** | | | | | | | **Domain 2: Index Test** | | | | | | **Domain 3: Reference Standard** | | | | **Domain 4: Flow and Timing** | | |
| --- | --- | --- | --- | --- | --- | --- | --- | --- | --- | --- | --- | --- | --- | --- | --- | --- | --- | --- | --- | --- |
|  | SQ1 | SQ2 | SQ3 | SQ4 | SQ5 | RoB | AC | SQ1 | SQ2 | SQ3 | SQ4 | RoB | AC | SQ1 | SQ2 | RoB | AC | SQ1 | SQ2 | RoB |
| Chen LD 2018 (35) | No | No | Yes | Yes | No | High | Low | Yes | Yes | Yes | Yes | Low | Low | Yes | Yes | Low | Low | Yes | No | High |
| Chen LD 2019 (36) | Yes | No | No | Yes | Yes | High | Low | Yes | Yes | U/C | Yes | U/C | U/C | Yes | U/C | Low | Low | U/C | Yes | U/C |
| Ding J 2021 (37) | Yes | No | Yes | Yes | Yes | High | Low | Yes | Yes | Yes | Yes | Low | Low | Yes | U/C | Low | Low | U/C | Yes | U/C |
| Ding J 2021 (38) | Yes | No | Yes | Yes | Yes | High | Low | Yes | Yes | Yes | Yes | Low | Low | Yes | U/C | Low | Low | U/C | Yes | U/C |
| Hu J 2020 (39) | Yes | Yes | Yes | Yes | Yes | Low | Low | Yes | Yes | Yes | Yes | Low | Low | Yes | Yes | Low | Low | Yes | Yes | Low |
| Kang HJ 2020 (40) | Yes | Yes | Yes | U/C | Yes | Low | Low | No | No | Yes | Yes | High | Low | Yes | U/C | Low | Low | Yes | No | High |
| Makoyeva A 2020 (41) | Yes | Yes | Yes | Yes | Yes | Low | Low | Yes | Yes | Yes | Yes | Low | Low | Yes | Yes | Low | Low | Yes | Yes | Low |
| Mulazzani L 2019 (42) | Yes | Yes | Yes | Yes | Yes | Low | Low | Yes | U/C | U/C | U/C | U/C | U/C | U/C | U/C | U/C | U/C | No | U/C | High |
| Pan JM 2021 (43) | Yes | Yes | Yes | Yes | Yes | Low | Low | Yes | Yes | Yes | Yes | Low | Low | Yes | Yes | Low | Low | Yes | Yes | Low |
| Polikoff A 2022 (44) | Yes | No | Yes | Yes | Yes | High | Low | U/C | U/C | U/C | Yes | U/C | U/C | Yes | U/C | Low | Low | U/C | Yes | U/C |
| Strobel D 2021 (45) | Yes | Yes | U/C | Yes | U/C | U/C | U/C | Yes | U/C | Yes | No | High | U/C | U/C | U/C | U/C | U/C | U/C | No | High |
| Terzi E 2017 (46) | Yes | Yes | Yes | U/C | Yes | U/C | Low | U/C | U/C | U/C | U/C | U/C | U/C | Yes | U/C | U/C | U/C | Yes | No | High |
| Zhou Y 2022 (47) | Yes | No | Yes | Yes | Yes | High | Low | Yes | Yes | Yes | Yes | Low | Low | Yes | U/C | Low | Low | U/C | Yes | U/C |

Summary: Risk of bias was most frequently rated as high in the domains of patient and observation selection and flow and timing, while index test and reference standard domains were generally rated as low risk.

Abbreviations: U/C, unclear.

**Table S2.** Positive Predictive Value (PPV) with 95% Confidence Intervals (CI) for P-values CEUS Major Feature Diagnostic Table Combinations (Figure 3) vs. Remaining Observations in the Overall LR Categories

| **LR-3 Diagnostic Table Combinations Vs. LR-3 Overall PPV Excluding Each Combination** | | | | | |
| --- | --- | --- | --- | --- | --- |
| **APHE** | **Size (mm)** | **Non-peripheral “washout”** | **Combination PPV (95% CI)** | **Overall LR-3 PPV (95% CI)** | **P-Value** |
| No APHE | <20 | No washout of any type | 27.4 (13.6-47.4) | 51.8 (42.5-61.0) | 0.07 |
| No APHE | ≥20 | No washout of any type | 39.0 (19.5-62.7) | 38.5 (27.1-51.2) | 0.68 |
| No APHE | <20 | Late and mild washout | 60.6 (43.4-75.6) | 40.0 (27.1-54.5) | 0.06 |
| APHE | <10 | No washout of any type | 100.0 (0.0-100.0) | 31.9 (14.3-56.7) | 1.00 |

| **LR-4 Diagnostic Table Combinations Vs. LR-4 Overall PPV Excluding Each Combination** | | | | | |
| --- | --- | --- | --- | --- | --- |
| **APHE** | **Size (mm)** | **Non-peripheral “washout”** | **Combination PPV (95% CI)** | **Overall LR-4 PPV (95% CI)** | **P-Value** |
| No APHE | ≥20 | Late and mild washout | 79.5 (64.1-89.4) | 69.3 (48.3-84.5) | 0.64 |
| APHE | ≥10 | No washout of any type | 69.2 (48.3-84.4) | 81.0 (66.3-90.2) | 0.53 |
| APHE | <10 | Late and mild washout | 100.0 (0.0-100.0) | 69.7 (49.7-84.3) | 1.00 |

**LR-5 Overall PPV vs. Diagnostic Table Combinations - only 1 combination**

Summary: Within LR-3 and LR-4 combinations, no statistically significant differences in PPV were observed between individual combinations and the overall category. Only one combination was present in LR-5. Comparisons were performed with the Wald Test.

Abbreviations: APHE, arterial phase hyperenhancement

**Table S3.** The Sensitivity Analysis of Positive Predictive Value (PPV) with 95% Confidence Intervals (CI) for P-values CEUS Major Feature Diagnostic Table Combinations (Figure #) vs. Remaining Observations in the Overall LR Categories

| **LR-3 Diagnostic Table Combinations Vs. LR-3 Overall PPV Excluding Each Combination** | | | | | |
| --- | --- | --- | --- | --- | --- |
| **APHE** | **Size (mm)** | **Non-peripheral “washout”** | **Combination PPV (95% CI)** | **Overall LR-3 PPV (95% CI)** | **P-Value** |
| No APHE | <20 | No washout of any type | 12.4  [0.6-76.4] | 45.03  [7.6-89.2] | 0.36 |
| No APHE | ≥20 | No washout of any type | 12.0  [0.0-100.0] | 30.3  [7.3-70.7] | 0.25 |
| No APHE | <20 | Late and mild washout | 100.0  [0.0-1.0] | 8.9  [0.04-96.0] | 1.00 |
| APHE | <10 | No washout of any type | - | - | - |

**LR-4 Overall PPV vs. Diagnostic Table Combinations - only 1 combination**

**LR-5 Overall PPV vs. Diagnostic Table Combinations - only 1 combination**

Summary: Within LR-3 combinations, no statistically significant differences in PPV were observed between individual combinations and the overall LR-3 category in sensitivity analysis. Only one combination was present in both LR-4 and LR-5. Comparisons were performed with the Wald Test.

Abbreviations: APHE, arterial phase hyperenhancement

**Table S4.** Chi-Square Test for CEUS LI-RADS Categories Individual Combinations

| **Chi-Square** | **LR-3** | **LR-4** | **LR-5** |
| --- | --- | --- | --- |
| **P-Value** | 0.02 | 0.02 | Only 1 LR-5 cell |

**Table S5.** Chi-Square Test for CEUS LI-RADS Categories Individual Combinations Sensitivity Analysis

| **Chi-Square** | **LR-3** | **LR-4** | **LR-5** |
| --- | --- | --- | --- |
| **P-Value** | 0.02 | Only 1 LR-4 cell | Only 1 LR-5 cell |

**Table S6.** Pairwise Comparisons using Bonferroni-Holm correction for the CEUS LI-RADS Categories Individual Combinations, see **Table S7** for Legend of Numerical Labeling.

| **LR-3** | | | |
| --- | --- | --- | --- |
|  | 1 | 2 | 3 |
| 2 | 0.63 | - | - |
| 3 | 0.51 | 0.63 | - |
| 5 | 0.12 | 0.63 | 0.63 |

| **LR-4** | | |
| --- | --- | --- |
|  | 4 | 6 |
| 6 | 1.00 | - |
| 7 | 1.00 | 1.00 |

Summary: Pairwise comparisons did not demonstrate any significant differences among LR-3 and LR-4 combinations. LR-5 is not shown here since there is only one combination. See Table S7 for legend.

Abbreviations. LI-RADS, Liver Imaging Reporting and Data System.

**Table S7.** Legend for **Table S6**, Pairwise Comparisons using Bonferroni-Holm correction for the CEUS LI-RADS Categories Individual Combinations

| **APHE** | **No Non-Rim APHE** | | **Non-Rim APHE** | |
| --- | --- | --- | --- | --- |
| **Size (mm)** | **<20** | **≥ 20** | **< 10** | **≥ 10** |
| **No washout of any type** | 1 | 2 | 3 | 4 |
| **Late and mild washout** | 5 | 6 | 7 | 8 |

LR-3 combinations (yellow), LR-4 combinations (orange), LR-5 combinations (red).

Abbreviations: APHE, Non-Rim Arterial Phase Hyperenhancement.

**Figure S1.** Sensitivity Analysis of Low-Risk Studies of the Positive Predictive Value (PPV) for Diagnosis of HCC with 95% Confidence Intervals (CI) for CEUS LI-RADS Major Feature individual Combinations

**
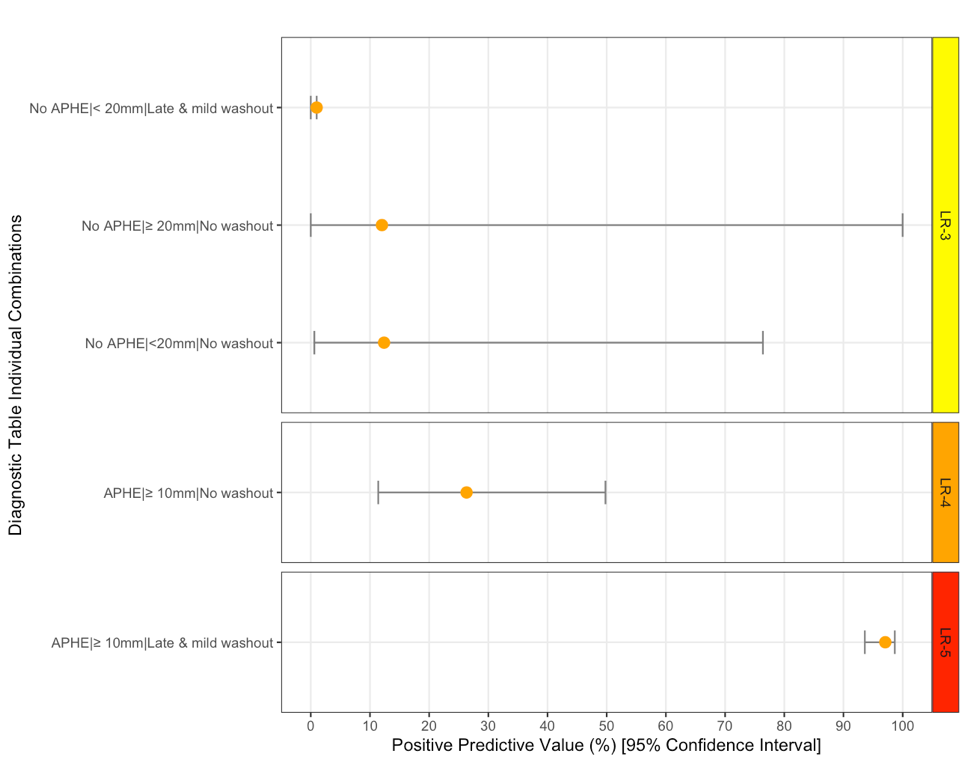
**

| Non-Rim APHE | Size (mm) | Nonperipheral Washout | Sample Size | I^2^  (%) |
| --- | --- | --- | --- | --- |
| No APHE | <20 | No NPW | 1 | 0.0 |
| No APHE | ≥20 | No NPW | 3 | 84.1 |
| No APHE | <20 | LM NPW | 2 | 0.0 |
| APHE | ≥10 | No NPW | 5 | 0.0 |
| APHE | ≥10 | LM NPW | 198 | 0.0 |
|  |  |  |  |  |

Summary: Random-effects model shows the sensitivity analysis of low-risk studies of the positive predictive values (PPVs) for diagnosis of hepatocellular carcinoma (HCC) for CEUS Liver Imaging Reporting and Data Systems (LI-RADS) major feature combinations compared with the LI-RADS grade overall PPV without the combination of interest. The individual feature combinations for the LI-RADS diagnostic table (left side) shows observation size (≥10 mm, <20 mm, and ≥20 mm), arterial phase hyperenhancement (APHE), and non-peripheral washout (NPW; no NPW or late and mild (LM) NPW). Between-study heterogeneity is quantified using I^2^, which reflects the proportion of variability due to heterogeneity rather than chance. The forest plot (right side) shows the PPVs (orange dots) with 95% CIs (gray lines). Combinations are grouped by LI-RADS category: LR-3 (yellow), LR-4 (orange), LR-5 (red). A total of 209 observations were included.

**Figure S2.** Diagnostic Table Illustration for **Figure S1**, the Sensitivity Analysis of Low-Risk Studies of the Positive Predictive Value (PPV) for Diagnosis of HCC with 95% Confidence Intervals (CI) for CT/MRI LI-RADS Major Feature Individual Combinations

| **PPV [95% CI]**  **I^2^**  **n** | **No Non-Rim APHE** | | **Non-Rim APHE** | |
| --- | --- | --- | --- | --- |
| **Size (mm)** | **<20** | **≥20** | **<10** | **≥10** |
| **No washout of any type** | 0.08 [0.002; 0.8]  0.0  1 | 0.3 [0.0; 1.0]  0.0  3 | - | 0.3 [0.1; 0.5]  0.0  5 |
| **Late and mild washout** | 1.0 [0.0; 1.0]  0.0  2 | - | - | 1.0 [0.9; 1.0]  0.0  198 |

Summary: The diagnostic table values used in Figure S1 are shown in the color-coded cells. Yellow cells represent LR-3, orange cells represent LR-4, red cell represent LR-5.

Abbreviations: APHE, arterial phase hyperenhancement
